# Supplementary material for: Directed attenuation to enhance vaccine immunity
Source: PLoS Comput Biol. 2021 Feb 1;17(2):e1008602. doi: 10.1371/journal.pcbi.1008602 (PMC7877766; doi:10.1371/journal.pcbi.1008602)
Supplement: S1 Text — We also present two alternative models for comparison to the basic model of the main text. (PDF) [file pcbi.1008602.s001.pdf]

S1 Text for Directed attenuation to enhance vaccine immunity

Rustom Antia, Hasan Ahmed, James J Bull

In this supplement, we use simulations of the dynamics of infection for insight into the interactions that give rise to the tradeoffs observed when we attenuate various immune-evasion pathways of the virus. The final part of this supplement also presents two alternative models for comparison to the basic model of the main text.

Dynamics of attenuation by modulation of innate immunity

Viruses can attenuate or evade innate immunity in a number of ways. In the basic model described by eqns (1), attenuation of innate immunity can occur by changes in 4 parameters: s_Z the rate at which innate immunity can be stimulated, ϕ_V the sensitivity of innate immunity to recognizing virus, d_Z the rate of waning of innate immunity, and k_Z the rate constant for killing of the virus by innate immunity.

The responsiveness of innate immunity can be increased by reducing the virus density at which innate immunity is triggered, ϕ_V, or by increasing the rate at which innate immunity is stimulated, s_Z. As seen in Fig 3, both types of changes yield a significant reduction in pathology. For the parameters chosen, an increase in s_Z does not bring about a substantial change in the final level of adaptive immunity, while a decrease in ϕ_V leads to a small reduction in the final level of adaptive immunity generated. The reasons for the changes in the final level of adaptive immunity is hard to intuit, but the feedbacks involved

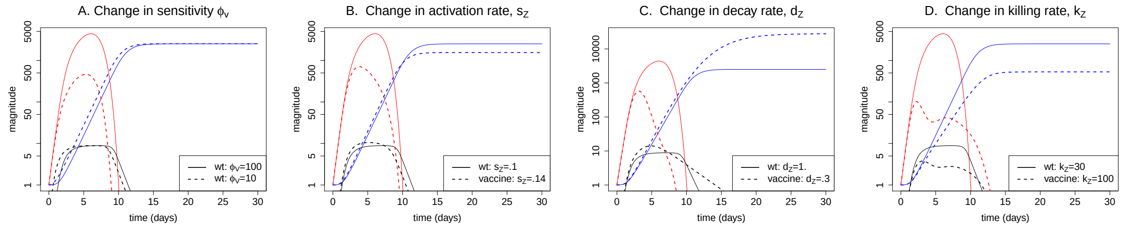

Figure S1. Dynamics of responses of vaccines that attenuate evasion of innate immunity. The plots show the dynamics of virus in red, innate and adaptive immunity in black and blue. The dynamics of the response to wild type virus is shown by the solid lines and the vaccine strain by the dashed lines. Parameters as in Table 1 except as indicated.

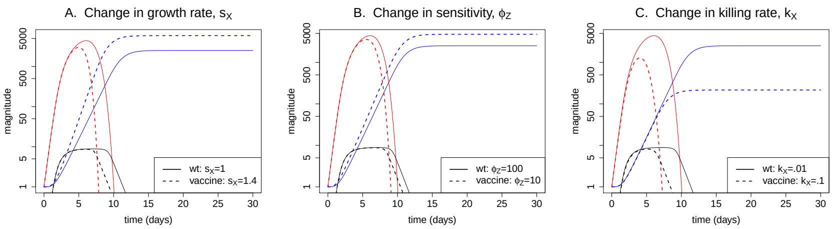

Figure S2. Dynamics of responses of viruses with enhanced levels of inducing and susceptibility to adaptive immunity. The plots show the dynamics of virus in red, innate and adaptive immunity in black and blue. The dynamics of the response to wild type virus is shown by the solid lines and the vaccine strain by the dashed lines. Parameters as in Table 1 except as indicated.

in determining the final level of adaptive immunity are illustrated by the representative simulations shown in Fig S1. While a decrease in ϕ_V or an increase in s_Z results in more rapid stimulation of innate immunity, the faster generation of adaptive immunity leads to more rapid control of the virus, which curtails the duration of expansion of the adaptive immune response. The final level of adaptive immunity depends on the magnitude of these two effects, the relative magnitudes of which we find hard to intuit without the help of the simulations. In the case of a decrease in ϕ_V, the more rapid stimulation of adaptive immunity is balanced by the shorter duration of stimulation of adaptive immunity, and thus the final level of adaptive immunity remains largely unchanged. In the case of an increase in s_Z, the duration of stimulation of the adaptive immune response is further curtailed by the more rapid clearance of the virus by innate immunity, and as a consequence this results in a lower final level of adaptive immunity.

Another way a virus can evade innate immunity is by increasing the rate of decay of innate immunity, d_Z. Again from Fig 3, a vaccine strain of the virus with a lower rate of decay of innate immunity than the wild type virus will not only reduce the extent of pathology but can even generate more immunity than is elicited by the wild-type vaccine strain. The cause of this can also be seen in Fig S1: the reduction in d_Z results in an extended duration for which innate immunity is above the threshold for stimulation of adaptive immunity.

The final way which the virus can evade innate immunity is decreasing the rate at which innate immunity kills the virus (k_Z). A vaccine strain of virus that has an increased susceptibility to innate immunity will have lower pathology and lower immunity (Fig 3). Fig S1 shows the cause – the virus is susceptible to killing and thus more rapidly controlled by the immune response. This results in a lower level of innate immunity and less stimulation of adaptive immunity.

Dynamics of attenuation by modulation of adaptive immunity

Viruses can attenuate or evade adaptive immunity in a number of ways. In terms of the parameters of the model, attenuation of adaptive immunity can occur by changes in 3 parameters: s_X, the maximum rate at growth of adaptive immunity; ϕ_Z, the sensitivity of stimulation of adaptive immunity to recognizing virus; and k_X, the rate constant for killing of the virus by adaptive immunity.

As seen in Fig 3, changes to the virus that increase the rate of proliferation of adaptive immunity, either by increasing the rate of proliferation of immune cells s_X or increasing the sensitivity of immune cells to stimulation ϕ_Z, result in attenuation (less pathology) and higher levels of immunity. The reason for this can be seen in Fig S2: increases in s_X or decreases in ϕ_Z result in faster / earlier growth of adaptive immunity. However faster generation of adaptive immunity results in more rapid clearance of the virus which leads to a slightly shorter duration of stimulation of the adaptive immune response. The net effect of these two factors acting in opposite directions is a slight increase in the final level of adaptive immunity.

As is the case for innate immunity, an increase in the rate of clearance of the virus by adaptive immunity (k_X) leads to more rapid clearance of the virus, and this results in a decrease in pathology but also a decrease in the extent of stimulation and final level of adaptive immunity (Fig S2).

Robustness

The key result in this paper is that targeting some but not all immune evasion pathways of the virus can lead to infections with reduced pathology but increased immunity compared to wild type. The presentation in the main text is based on a single model described by eqns (1); here we examine the robustness of directed attenuation to changes in model structure. Two variants of our model are considered: (Alternate Model 1) the adaptive immune response depends only on the amount of virus antigen; (Alternate Model 2) the stimulation of adaptive immunity depends on both the activation of innate immunity and the amount of virus antigen. The equations for virus and innate immunity are unchanged.

$$\frac{dX}{dt} = s_X X \left(\frac{Z}{\phi_Z + Z}\right) \qquad \text{(Basic model from main text)}$$

$$\frac{dX}{dt} = s_X X \left(\frac{V}{\phi_X + V}\right) \qquad \text{(Alternate Model 1)}$$

$$\frac{dX}{dt} = s_X X \left(\frac{Z}{\phi_Z + Z}\right)\left(\frac{V}{\phi_X + V}\right) \qquad \text{(Alternate Model 2)}$$

Fig S3 shows trade-off plots corresponding to those in Fig 4 of the main text. Directed attenuation is possible for both of these Alternate Models, and indeed, is possible for several

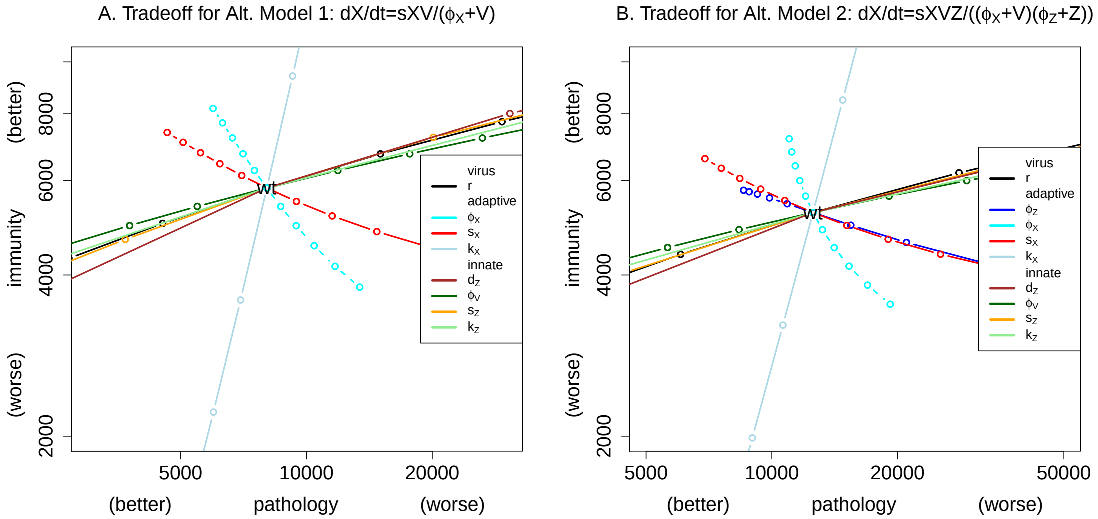

Figure S3. Directed attenuation is feasible with alternative models. This figure illustrates tradeoffs between pathology and immunity for Alternate Models 1 and 2 in panels A and B, respectively, for comparison to the tradeoffs with the basic model in Fig 4. Patterns differ somewhat between the two panels as well as between this figure and Fig 4 either because a parameter in one model is not an element of another model (e.g., ϕ_X, ϕ_Z) or because the parameter does not have the appropriate effect in all models (d_Z). Parameter values are as in Table 1 except $k_X = .001$ (to compensate for the lack of expansion when the virus is cleared compared with the basic model), and $\phi_X = 10^2$.

of the same parameters as with the basic model (note that some parameters used in the basic model are not components in these Alternate Models). These results indicate that the possibility of directed evolution is not limited to the basic model of this paper.
