# Supplementary material for: Directed attenuation to enhance vaccine immunity
Source: PLoS Comput Biol. 2021 Feb 1;17(2):e1008602. doi: 10.1371/journal.pcbi.1008602 (PMC7877766; doi:10.1371/journal.pcbi.1008602)
Supplement: Attachment — Submitted filename: PlosResponseToReviewers.rtf [file pcbi.1008602.s002.rtf]

Dear Editor and Reviewers,

Thank you for the reviews and suggestions. They have helped us revise and improve the manuscript.  The changes made are described below together with the reference to their  locations in the manuscript and are highlighted in the version marked highlighted. The included line numbers pertain to the file named Related Manuscript (smartVaccinePLOS_V2-highlighted.pdf) which has the changes highlighted and includes the figures embedded in the document and not the file formatted per the PLOS LaTeX template.

The detailed list of your responses to all review comments, and a description of the changes you have made in the manuscript are below.

Thanking you, 

Yours sincerely,

Rustom Antia, Hasan Ahmed and James Bull.

Changes made in response to reviewers comments. (responses in blue)

Reviewer #1: This is a well-written paper presenting a novel and interesting idea on the more intelligent design of vaccines. The idea is illustrated by analyzing an elegantly simple model of the innate and adaptive immune system. This modeling is required as results are surprising: in retrospect most parameter combinations that work are intuitive, but I would not have been able to predict these particular combinations beforehand. I also like your figures: very intuitive and clear.

I have only two major recommendations:
1. One can test the generality of the main results, i.e., the 3 parameter combinations giving decreased pathology and increased immunity, by exploring a few alternative models. For instance, a model where the adaptive immune response is (also) limited by antigens.

Response: We have explored the generality for two alternative models. As suggested by the reviewer in the first alternative model the proliferation of adaptive immune response is dependent on the antigen (and not innate immunity) and in the second the proliferation of the adaptive immune response is dependent on BOTH the antigen level AND the activation of innate immunity.  For both of these alternative models we get a qualitatively similar result insofar as attenuation of some but not all immune evasion pathways of the virus results in a decrease in pathology AND an increase in the final level of adaptive immunity.  This is discussed in Section 3.5 (lines 217-228) and the details of the models and the plots of the results are in the SI (lines 563-575 and Figure 8).  

2. It would be good to see if the main mathematical results can be put into intuitive general conclusions like: prolonging innate immune is beneficial because it extends the period over which the adaptive immune response expands.

Response: We have attempted to do so. Briefly:
1. The results are intuitive for changes in the killing rates kz and kx for innate and adaptive immunity.  An increase in kz and kx (virus which does not avoid killing by immunity) results in more rapid virus clearance and thus a lower duration of stimulation and final level of adaptive immunity.  
2. The results are not intuitive for changes the parameters s_z, s_x, phi_z and phi_x that affect the magnitude of innate or adaptive immunity. This is because changes in these parameters have multiple effects  working in opposite directions.  The first is the more rapid generation of immunity. The second effect arises because the more rapid generation of immunity results in faster clearance of the virus, and this curtails the duration of stimulation of adaptive immunity.  The net result is thus hard to intuit. 
3. As referee 1 correctly points out the predominant effect of a decrease the parameter d_z is a prolongation of the stimulation of adaptive immunity. 

We have described these effects in the paragraph lines 241-264 and also in the SI lines 517-543 and 550-562

Minor comments:

Page 2: Historically, the mechanism of attenuation has been one of genetically reduced viral growth rate,
=> Historically, the mechanism of attenuation has been a reduced viral growth rate, ?

Corrected and highlighted

Page 3: (e.g. for mumps where the and necessitates periodic ..
something missing

Corrected and highlighted lines 44-45.

Page 4: so Z equals the fraction of the the maximum
the the

Corrected and highlighted line 76

Page 4: It is justified on biological grounds that the generation of immune responses requires stimulation by innate immunity and that this can occur even after replicating antigen is cleared.
Please provide references for this.

Thank you for pointing out this.  We had not been clear and have re-written this and added references (lines 79-84). 

It is justified on biological grounds that the generation of an adaptive immune response requires stimulation by activated innate immunity (e.g. a danger signal); the observation that virus antigen is typically presented by antigen-specific immune cells in in the lymph nodes for an extended period, potentially after live virus is cleared, also supports disconnecting adaptive immunity expansion from live virus [26, 27, 28, 29]

Table 1: Explain the 10^2v .02z: what is v and z?

v represents the units of virus and z of innate immunity.  We have indicated this in the legend to Table 1.

Page 10: However if an individual was To overcome this problem
lower case T

Corrected.

Page 15: However they (i.e. [57]) suggested
Write our the they

Corrected on line 305

Finally, although I agree that this needs to be worked our for every particular virus, filling in these details and modeling them in an intuitive manner will be a major challenge. Again, it would be good to have a better sense of the generality of the results.

Reviewer #2: This is a very interesting paper, which proposes parameters of the interaction between a pathogen and the immune system which could be targeted in designing a vaccine. The authors suggest that making various changes to these parameters, such as extending the lifespan of innate immunity, could simultaneously lower the pathology of the vaccine and raise immunity to it. Standard vaccines involve a pathogen whose growth rate is diminished. This results in lower pathology, but also lower immunity. The effects of simultanously varying various pairs of parameters of the model of pathogen and immune response are attractively summarised in heat maps of the pathology and immunity. If it is found possible to vary the identified parameters biologically, this could be very useful in designing vaccines.

Table 1 should be referred to in the text and authors should explain how their parameter estimates are derived.

Done on pages 4-5 lines 85-95.  

There are also a few tiny errors:
p3 "This can potentially result in reinfection with circulating virus (e.g. for mumps where the and necessitates periodic boosting[12, 17]." This sentence is incomplete.

Corrected and highlighted lines 44-45.

p4 "The approach to directed attenuation presented here is to develop a model that incorporates key elements of out current understanding of ..." (insert "of")

p10 "However if an individual was to" ("To" -> "to")

Corrected.

p22 "As seen in Fig 3, in both these scenarios.." ("In" -> ", in"; "scenario's" -> "scenarios")

Corrected.

p 22 "As the adaptive immune response is needed for virus clearance we find that the duration of infection is not significantly affected and consequently the amount of adaptive immunity generated does not substantially decrease." (add "decrease")

Corrected (this paragraph has been re-written).

 
